# Programmatic factors associated with the limited impact of Community-Directed Treatment with Ivermectin to control Onchocerciasis in three drainage basins of South West Cameroon

**DOI:** 10.1371/journal.pntd.0005966

**Published:** 2017-11-20

**Authors:** Christian Tetteh Duamor, Fabrice Roberto Datchoua-Poutcheu, Winston Patrick Chounna Ndongmo, Aldof Tah Yoah, Ernest Njukang, Emmanuel Kah, Mary Sheena Maingeh, Jonas Arnaud Kengne-Ouaffo, Dizzle Bita Tayong, Peter A. Enyong, Samuel Wanji

**Affiliations:** 1 Epidemiology and Control of Infectious Diseases, Department of Microbiology and Parasitology, University of Buea, Buea, Cameroon; 2 Accra School of Hygiene, Ministry of Health, Korle-Bu, Accra, Ghana; 3 Research Foundation for Tropical Diseases and Environment, Buea, Cameroon; Imperial College London, UNITED KINGDOM

## Abstract

**Introduction:**

The CDTI model is known to have enhanced community participation in planning and resource mobilization toward the control of onchocerciasis. These effects were expected to translate into better individual acceptance of the intervention and hence high Treatment Coverage, leading to a sustainable community-led strategy and reduction in the disease burden. A survey revealed that after 10–12 rounds of treatment, prevalence of onchocerciasis was still high in three drainage basins of South West Cameroon and transmission was going on.

**Methods:**

We designed a three (3)-year retrospective (2012, 2013 and 2014), descriptive cross-sectional study to explore the roles of operational challenges in the failure of CDTI to control the disease as expected. We administered 83 semi-structured questionnaires and conducted 12 in-depth interviews with Chiefs of Bureau Health, Chiefs of Centers, CDDs and Community Heads. Descriptive statistics was used to explore indicators of performance which were supported with views from in-depth interviews.

**Results:**

We found that community participation was weak; communities were not deciding time and mode of distributions. Only 6 (15.0%) of 40 Community Drug Distributors reported they were selected at general community meetings as required. The health service was not able to meet and discuss Community-Directed Treatment with Ivermectin activities with individual communities partly due to transportation challenges; this was mostly done through letters. Funding was reported to be inadequate and not timely. Funds were not available to conduct Community-Self Monitoring after the 2014 Mass Drug Administration. There was inadequate health staff at the frontline health facility levels, and some Chiefs of Center reported that Community-Directed Treatment with Ivermectin work was too much for them. The mean operational Community Drug Distributor-population ratio was 1 Community Drug Distributor per 317 populations (range: 194–464, expected is 1:250). Community Drug Distributor attrition rate was 14% (2012), 11% (2013) and 12% (2014) of total Community Drug Distributors trained in the region. Lack of incentive for Community Drug Distributor was primary reason for Community Drug Distributor attrition. Number of Community Drug Distributors trained together by health area ranged from 14 to 127 (mean ± SD = 51 ±32) with duration of training ranging from 4–7 hours (mean ± SD = 5.05 ± 1.09). The trainings were conducted at the health centers. Community Drug Distributors always conducted census during the past three distributions (Mean ± SD = 2.85 ± 0.58). Community-Self Monitoring was facing challenge. Several of the community heads, Chiefs of Bureau Health and Chiefs of Center agreed that Community-Self Monitoring was not being carried out effectively due to lack of incentives for monitors in the communities.

**Conclusion:**

Inadequate human resource, funding issues and transportation challenges during distribution periods reduced the ability of the health service to thoroughly sensitize communities and supervise CDTI activities. This resulted in weak community understanding, acceptance and participation in the process. CDTI in our study area did not achieve sustainable community-led campaign and this may have led to the reduced impact on Onchocerciasis.

## Author summary

River blindness is caused by a very tiny, thread-like worm. The disease is better controlled when affected communities are included in the planning and carrying out of distribution of Ivermectin used to treat the disease. For a community to be able to prevent people from getting this disease, members must take Ivermectin once or twice a year, continuously for about 20 years. Hence, the organization in charge of controlling river blindness (African Programme for Onchocerciasis Control–APOC) decided that when a control programme is started in a community, the community must be involved and assisted to take full charge of the programme so that within 12 years the community can sustain the distribution of Ivermectin for as long as necessary. This community directed strategy prevented river blindness in many communities. However, after 10–12 years of implementation, studies found that river blindness largely persists in communities in three drainage basins in South West Region of Cameroon. This paper discussed the operational challenges that the programme may have faced in these areas.

## Introduction

The Community-Directed Treatment with Ivermectin (CTDI) approach adopted by the African Programme for Onchocerciasis Control [1] accelerated progress towards control of Onchocerciasis through increased community participation and higher treatment coverage of affected populations [2,3]. The CDTI model is known to be cost-effective, fosters high level of community acceptance and empowerment [4]. The success of the CDTI process has been attributed to the nature of collaboration among the stakeholders. The stakeholders involved in the CDTI process are some 146 000 affected communities, health services (ministries) of participating countries, Non-Governmental Developmental Organizations (NDGOs) and external donor countries and organizations [5–7].

The partners have specified roles relating to planning, resourcing, drug distribution, monitoring and evaluation, social mobilization and training of implementers: The NDGOs and external donors are responsible for providing technical/operational support, funds for logistics, training and monitoring as well as ensuring that Ivermectin gets to national offices of the programme [6]. The governments, through the ministries of health are responsible for approaching and sensitizing communities, training of implementation staff, supervision of the process and ensuring an adequately motivated staff. APOC prescribed the required number of training, supervision, monitoring and review meetings to be carried out at each level of the health service annually [7]. The communities are responsible for selecting community drug distributors (CDDs) and deciding how to motivate them, plan when and how to distribute Ivermectin, distribute the drug and ensure members adhere to treatment, conduct Community Self-Monitoring (CSM) and report to the health service [8].

**Fig 1:**
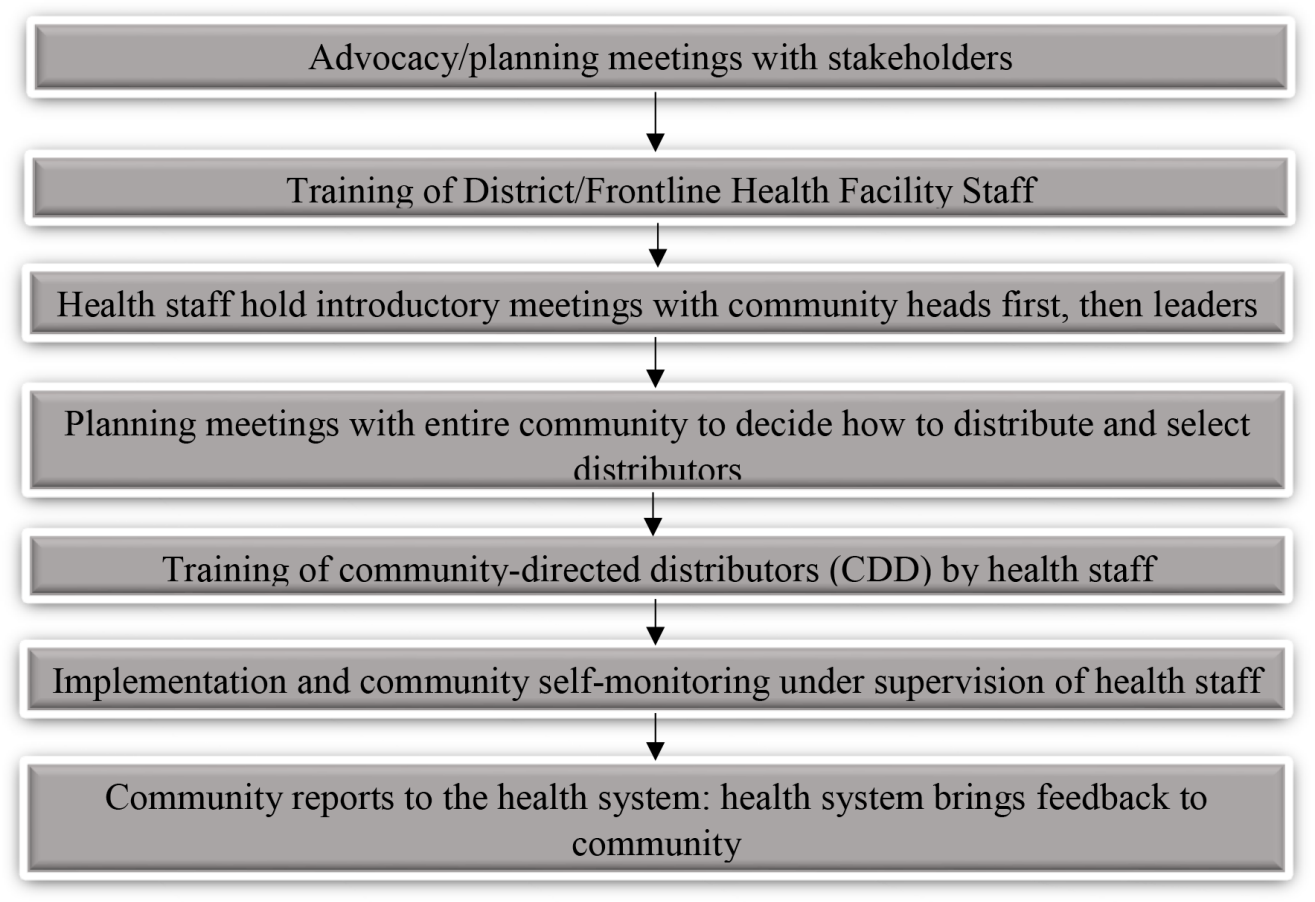
Organogram of CDTI establishment. Adapted from (APOC/WHO, 1998).

Central to the CDTI process is the maximized structural community participation [1,9,10]. To win the trust of communities and build strong partnership however, the health service must carry out series of advocacy meetings (Fig 1) [8]. This requires adequate and well-resourced health staff at the front line health facility levels.

Ivermectin mass drug administration process involves annual training of staff, census to determine drug needs of communities, distribution of the drug, conduction of CSM and reporting to the health services, the health service providing feedback to communities in addition to supervision and review meetings. These activities mandate adequate and timely availability of human, material and financial resources to ensure successful implementation. This manuscript reviewed the implementation process during the 2012, 2013 and 2014 distributions.

### Cameroon health system

Cameroon health system is coordinated by a Ministry of Public Health headed by a Minister, who works through ten (10) Regional Delegations of Public Health headed by medical doctors. A region is divided into Health Districts, headed by district medical officers who are also medical doctors; there are eighteen (18) health districts in the South West Region. A health district is further divided into health Areas. A health area comprises about 5–10 communities that are served by a health center which may have smaller health posts in addition. The health center is usually headed by a senior nurse called Chief of Center. A nurse in charge of a health post is called Chief of Post. Health decisions at this level are taken by a Health Area Committee. This is the level where the health system interacts with communities, also referred to as frontline health facilities. In CDTI, CDDs collect Ivermectin from here and report directly to health personnel at the frontline health facilities.

A community or village in Cameroon is headed by a chief or community head. The community is demarcated into what is known as Quarters, comprising of about twenty (20) houses depending on the size of the community. Each quarter has a head who takes decisions with household heads of all households in the quarter. The quarter heads and community head forms the village council. They oversee the work of CDDs, other developmental activities and resources in the community. Decisions are disseminated by quarter heads through household heads or through an announcer called ‘town crier’. However, the influence of these traditional rulers varies among the tribes and is generally eroding away very fast.

The implementation of CDTI in Cameroon started in 1998. There are currently 15 CDTI projects in Cameroon (the third largest after Nigeria and DR Congo). The two CDTI projects, South West I & II situated in the South West Region are responsible for reaching some 1,367 endemic communities with Ivermectin [8]. They were approved in 1998 and 1999, and became operational in 1999 and 2000 respectively. The process started in 1999 in communities of the Meme and Mungo river drainages, and in the year 2000 in communities of the Manyu river drainage (South West Regional Delegation of Health Database).

South West CDTI Project I covers the Meme and Mungo drainage basins. It achieved 100% geographic coverage from 2001–2014, except in 2008 (97%) and 2009 (98.33%). Therapeutic coverage rose from 32.56% in 2001 to 82.83% in 2010, and has since achieved at least 81% as at 2014. The South West CDTI Project II, which covers the Manyu drainage basin, achieved a geographic coverage between 95% - 100% from 2001–2014. Therapeutic coverage rose from 37.2% in 2001 to 83.7% in 2009, the project has since achieved closely same coverage as at 2014.

According to ONCHOSIM predictions, the outcome of elimination of onchocerciasis depends on precontrol endemicity level, frequency of MDA and treatment coverage (TC) achieved [11]. Given the precontrol hyperendemic (60–98% prevalence) levels in communities in our study area, CDTI projects here must achieve and sustain annual TCs above 80% in order to bring the disease under control in about 18 years [11]. Kim *et al*. predicted that the elimination scenario for onchocerciasis is feasible by 2028 in some areas but could go beyond 2045 in countries with operational challenges [12]. Certain challenges have been identified with the CDTI process. These include: maintaining timely drug-collection mechanisms; integrating CDTI with existing primary-healthcare services; strengthening local health infrastructure; achieving and maintaining an optimal treatment coverage; establishing and scaling up community self-monitoring; designing and implementing operational research locally; ensuring the adequacy of community-directed distributors; increasing the involvement of local non-governmental developmental or community-based organizations in the programme; achieving financial sustainability; implementing equitable cost-recovery systems; and engaging in effective advocacy among the stakeholders, especially with the affected communities [13].

After twelve (12) annual MDA with Ivermectin in communities in three drainage basins in the South West Region of Cameroon, a situation analysis of onchocerciasis through entomological and parasitological (epidemiological) surveys revealed that prevalence and transmission did not reduce as predicted (Prevalence of microfilaria: Meme drainage basin– 52.7%, Mungo drainage basin– 41.0%, Manyu drainage basin– 33.0%) [14,11]. This paper explored the possible roles operational challenges could have played in the failure of CDTI to control the disease in these areas, by examining the implementation practices over the past three distributions (2012–2014).

### Specific objectives

We carried out this study to document the programmatic factors associated with the limited impact of CDTI to control onchocerciasis in three drainage basins. We also wanted to assess the community perceptions as well as their level of participation in the CDTI process.

### Hypothesis

We hypothesized that stakeholders were not able to fully implement the CDTI protocols and hence communities did not adhere adequately to treatment.

## Methods

### Study site

The study was carried out in five (5) health districts located in three forest drainage basins of South West Region of Cameroon. These drainage basins receive about eight (8) months of rainfall and hence create long periods of favorable ecological environment for the insect vector (blackflies). About 90% percentage of the road network in the drainage basins were not tarred. The poor road network, coupled with long rainy seasons pose transportation challenges to health supervision teams.

### Study population

The respondents were the South West Regional Coordinator for Neglected Tropical Diseases (NTDs), the Chiefs of Bureau Health (CBH) of five (5) Health Districts (1 female and 4 males), 12 Chiefs of Centers (COC) (12 Health Areas; 8 females and 4 males), 40 CDDs (7 females and 33 males; 30 farmers; 34 married; all had at least primary school leavers’ certificate) and 24 Community Heads from communities across the five health districts. Once a community was included in the study, its head and CDDs were sampled. The COCs and CBHs of the health areas and health districts of the community also become part of the respondents. Since communities were randomly selected, bias was reduced.

### Design

A 3-three year retrospective, cross-sectional and descriptive approaches were used to explore possible weak links in the collaboration of the stakeholders and context specific factors that may be acting as implementation barriers to the CDTI process in the these drainage basins.

### Measurements

Data was collected with standard, semi-structured questionnaires and guides for in-depth interviews developed with reference to prescribed functions of stakeholders in the CDTI process [4,8,15]. Twelve (12) in-depth interviews were held with a community head, CDD, COC, and CBH from each drainage basin and also with the regional coordinator for NTDs. The interviews were held in English, recorded with a 4GB capacity Xgenx digital voice recorder (GDVR-901) and were transcribed into Microsoft word text format.

Performance of CDTI implementers were measured as number of training, census, supervision, CSM, reporting and review meetings conducted during the past three distributions (2012–2014). Adequate and timely arrival of funds and drugs, adequate health workers, amount of sensitization done, community participation as well as physical challenges to carrying out of the process were also assessed. Data collection was done from May to July, cleaning and analysis was done during August and September, 2015.

### Data analysis

Quantitative information was entered into a template created in Epi info version 3.5.4. The data was imported into excel and cleaned. It was then exported to SPSS version 20 and analyzed. Descriptive measures were used to explore indicators of good performance of the CDTI process. All statistical differences were considered significant at p < 0.05.

The in-depth interviews were transcribed verbatim and translated where necessary into English by a trained transcriber into separate Microsoft word documents, through the familiarization process. The transcripts were imported into Atlas.Ti, transformed into rich text format (rtf) and given file names recognizable by Atlas.Ti. The framework approach was used to screen the data following the development of an initial thematic framework. The transcripts were then coded following the emerging themes previously identified to retrieve meaningful views expressed by our informants. Frequently expressed views were used to explain and to compare with observed trends in the quantitative data.

### Ethics statement

Ethical approval was obtained from the Institutional Review Board, Faculty of Health Science, University of Buea. Administrative authorization was obtained from the South West Regional Delegation of Public Health. Administrative authorizations were also obtained from health districts after thorough review of the study protocol. The objectives, importance and ethical provisions of the study were explained to the respondents and informed verbal consent was obtained before the questionnaires and in-depth interviews were administered.

## Results

### Community participation

The communities were participating in CDTI mainly by selecting CDDs and individual members were giving cash incentive to CDDs. All the health personnel interviewed stated that the communities were yet to appropriate the CDTI concept. One CBH stated: “*APOC and ministry of public health and the rest I can grade them to be good*, *not very good*. *Is the community that is bringing weakness in the partnership*”.

**Fig 2:**
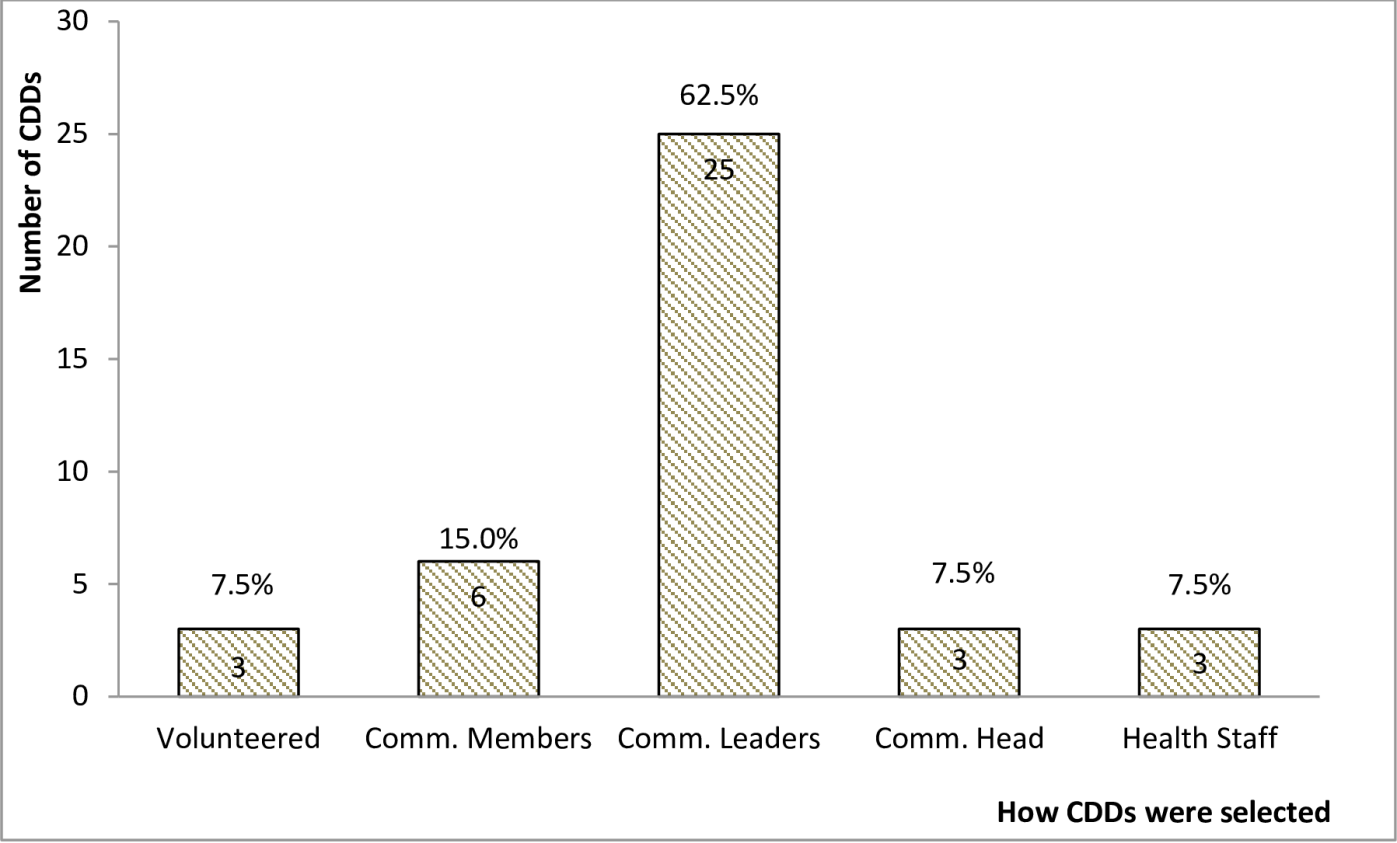
Mode of selection of CDDs in the study communities.

Distribution activities were planned top-down. All 24 community heads reported they did not participate in deciding the period (time of year) and the mode of distributing Ivermectin; whether by door-to-door or at a central location. This was confirmed by the statement of another CBH: “*I can say we have not really […] involved them in the planning and when you go and plan something like that and come to tell people without involving them*, *they become reluctant to participate*”. The communities were however given the opportunity to choose their CDDs and decide how to motivate them. Out of 40 CDDs sampled, 25 (62.5%) reported having been selected at a meeting of community leaders, 6 (15.0%) at a meeting of all community members and 3 (7.5%) by health workers (Fig 2).

### Sensitization of communities by the health service

The primary media used in creating awareness of CDTI activities among the communities included TV/Radio announcements at the regional level; banners, posters, badges (T-shirts and caps for CDTI personnel and key community members) at district and health area levels; at community levels, it was done through CDDs, town criers and religious groups. Review of the sensitization records showed that over the past three distributions (years), 2 TV announcements (alongside news scrow during the distribution weeks), 131 radio announcements, 9 872 posters and 11,300 fliers (smaller posters) were used to sensitize communities in our study area which had a population of 546 136 (Table 1). The posters and fliers were normally pasted at key public places such as health centers or given to CDDs and other key community members.

**Table 1:** Mode of sensitization on Ivermectin distribution over the past three years in the health districts of the study sites.

Health District	Medium of sensitization			
	TV	Radio	Posters	Fliers
**Kumba**	NA	-	-	-
**Mbonge**	NA	0	-	-
**Mamfe**	NA	0	592	900
**Konye**	NA	0	300	700
**Eyumujock**	NA	0	480	800
**Sub-total**	0	0	1 372	1 400
**Regional level**	2 + News scrow	131	8 500	8 900
**Grand total**	2	131	9 872	10 300

NA = Not applicable

**Fig 3:**
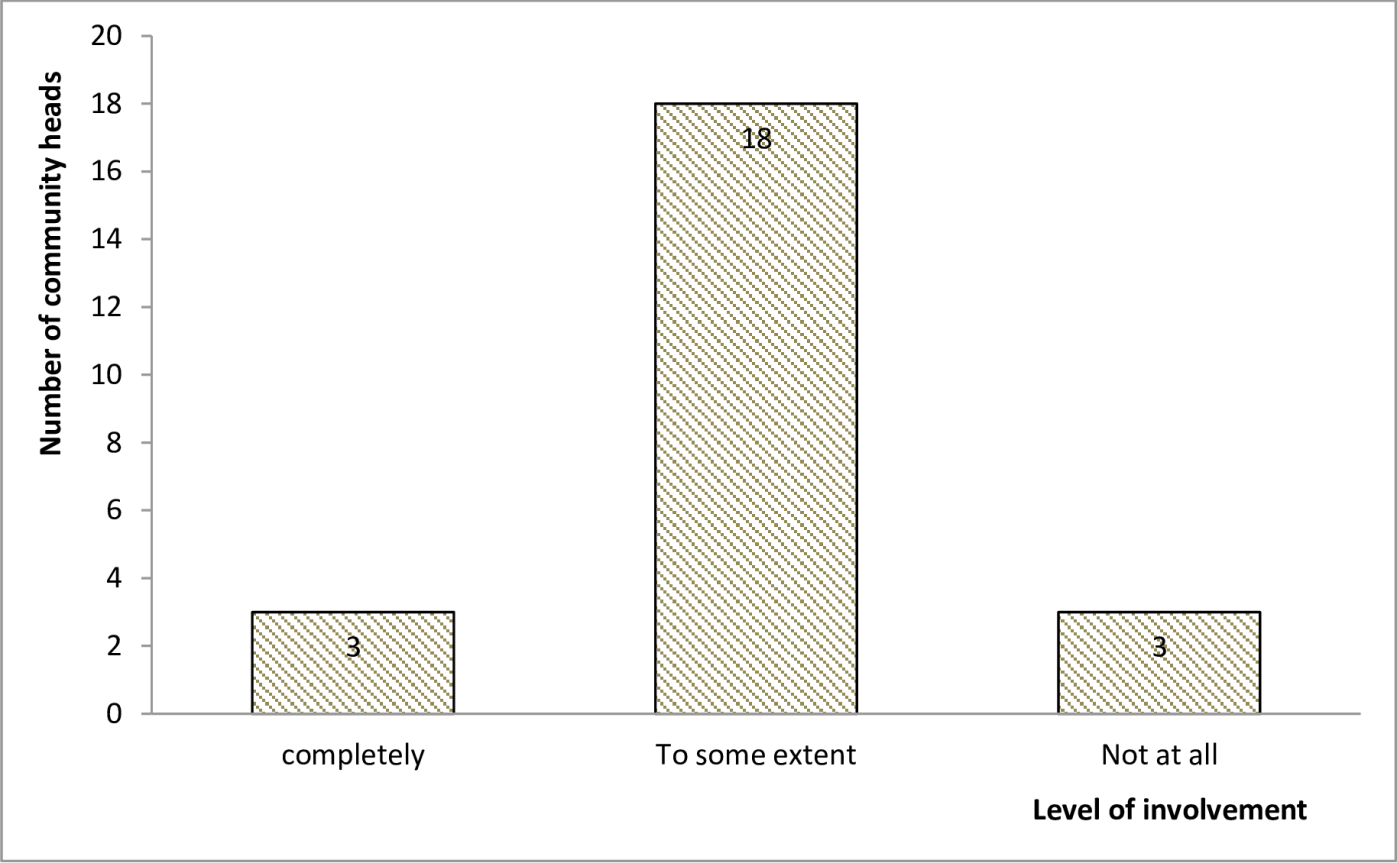
Different levels of involment of community heads in sensitization.

**Fig 4:**
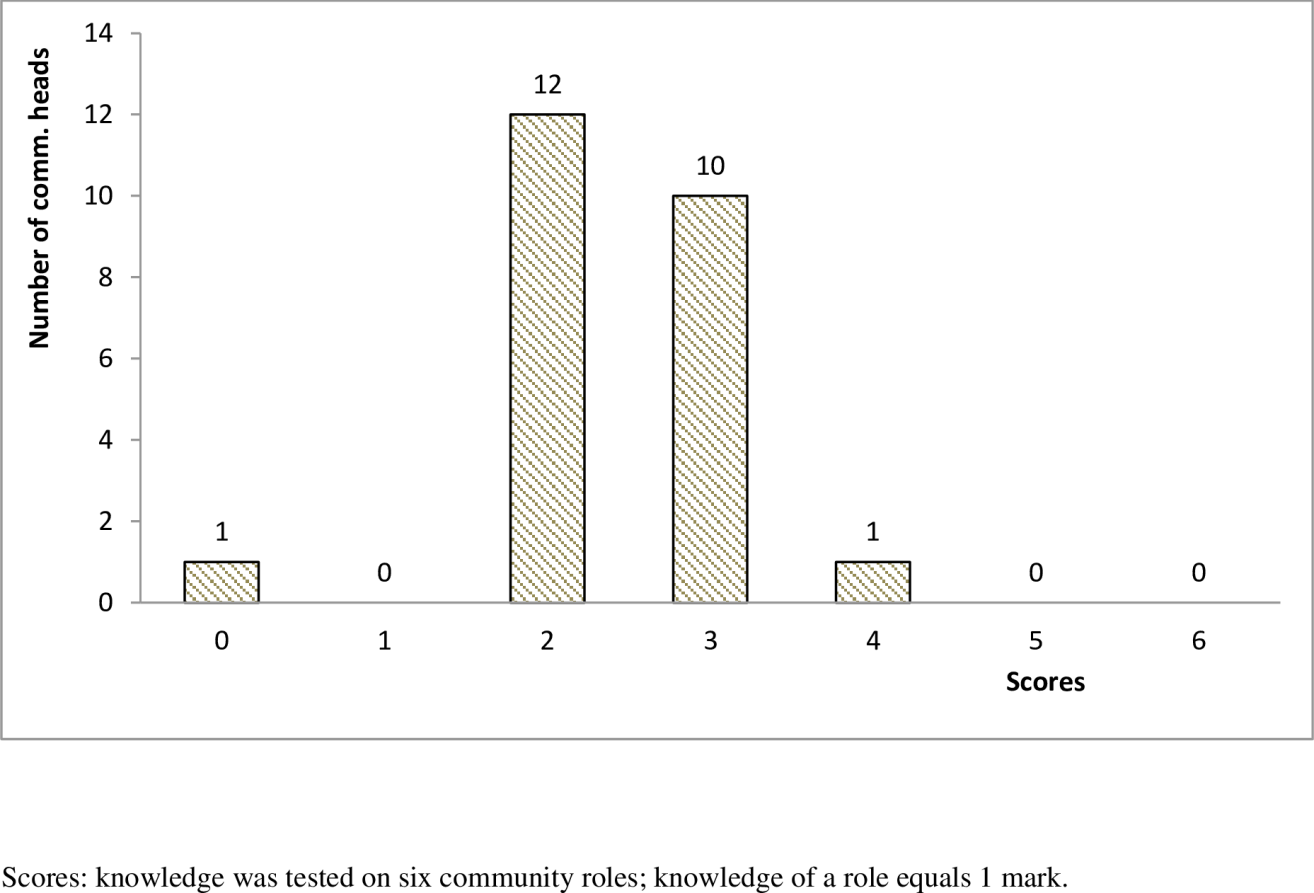
Level of Community heads awareness of community roles in the CDTI process.

Only three (3) out of twenty-four (24) community heads reported having been completely involved in the sensitization of their members (Fig 3). Instead of the personal visit to the head of communities and their leaders, health areas were informing them about distribution through letters. One community head reported: *“I only hear that [*…*] is where they deposit the drugs and other health units go for it*. *That’s all the information that I know*, *but for the […] other issue that they will call or they bring the drugs and the community now […] decides on how to distribute it*, *to me we have never have such information”*. The CBHs and COCs also admitted that the communities have not been fully sensitized on their roles. The view of one COC is presented as follows: *“I just know*, *I think that we have not really sensitized them well*, *we have not really involved them well*. *They do not know their duties […]*. *And so I think that the weakness is our own”*. One CBH also pointed out: *“The second thing I will say is the failure of the health personnel to sensitize the community members to actually understand what their role is as partners”*. The scores by the community heads based on their knowledge of communities’ roles as stakeholders in CDTI are shown in Fig 4.

### Mobilization of resources for CDTI process

#### Financial resource

Funding for training, supervision, monitoring and other aspects of CDTI was being provided by Non-Governmental Developmental Organizations (NGDOs like Sightsavers International–SSI, Hellen Keller International—HKI)—The main implementing partner for CDTI in our study area was Sightsavers and external donors like (United States Agency for International Development—USAID, WHO and APOC). Funds were released in phases, justification of use of previous funds must reach the funders before they release funds for the next activity. According to a CBH, sometimes the funds delay: “*I will say it comes not very on time*. *As am talking to you now*, *Mectizan is already at the health areas and we are waiting for funds for CDD training then they kick off the exercise”*. A COC reported: “*we have to pre-finance CDD training*, *when the money comes then they pay the health center”*.

**Fig 5:**
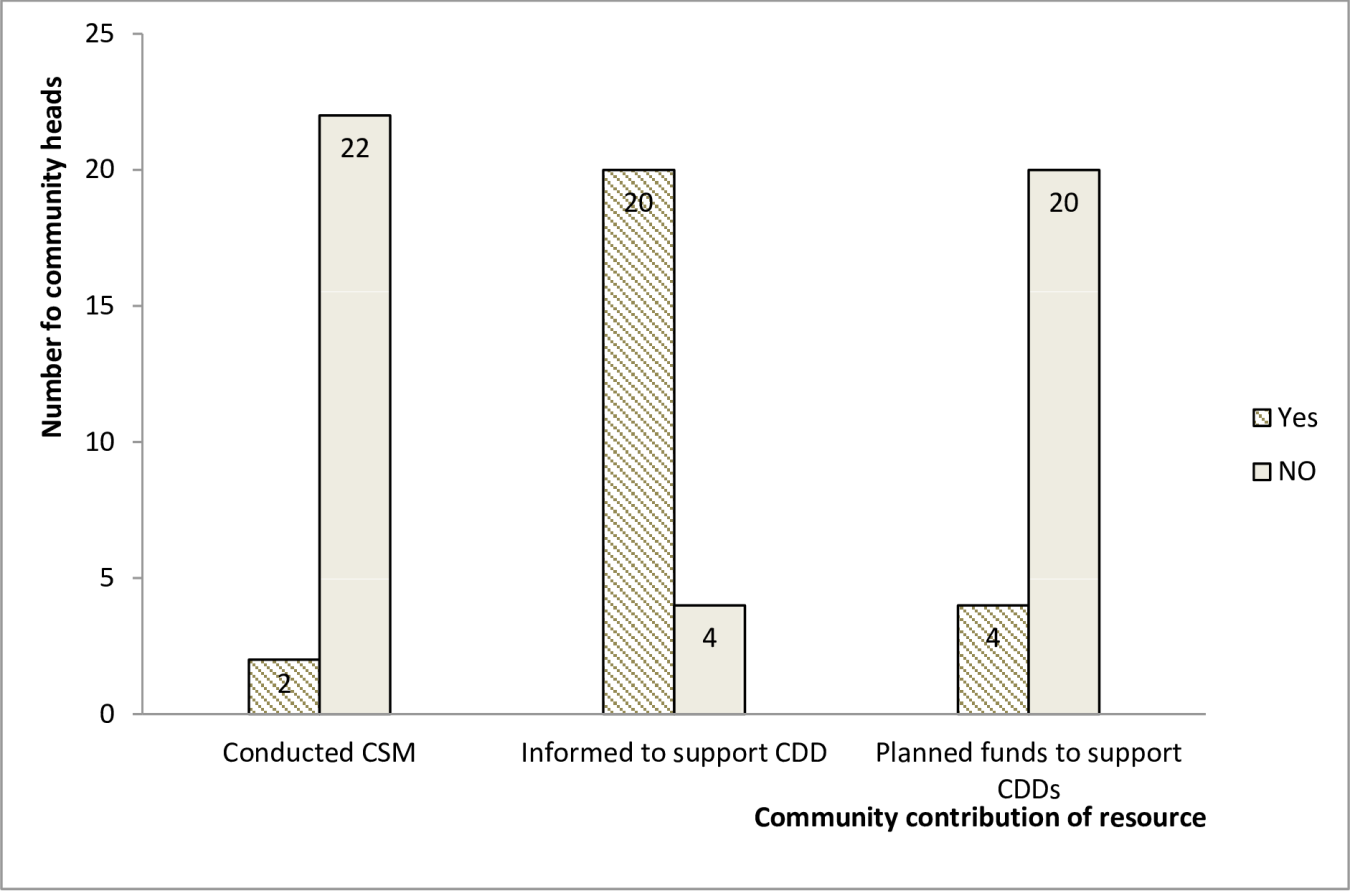
Community heads awareness and contribution.

**Fig 6:**
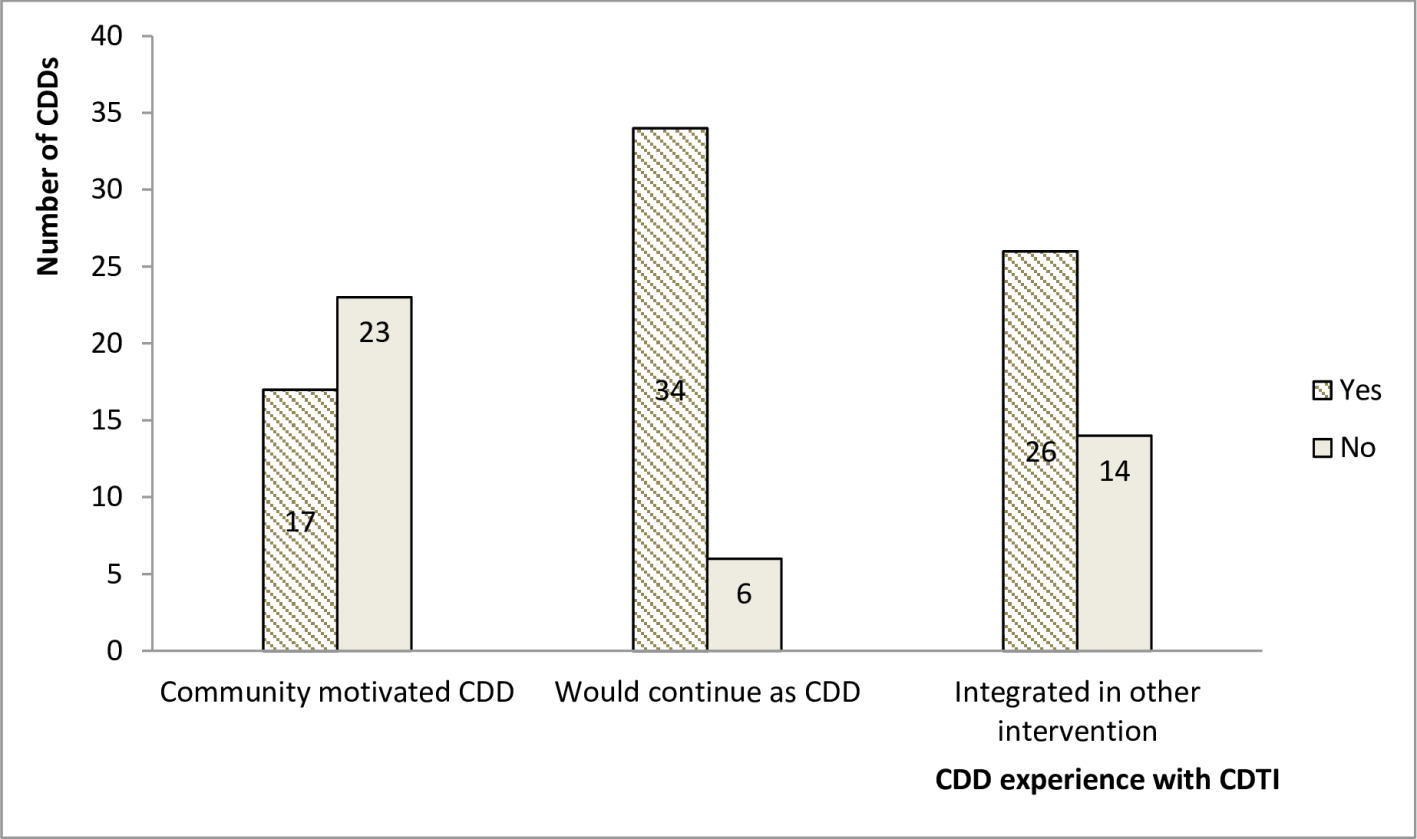
The experiences of 40 CDDs with CDTI process.

The average percentage of government budget for CDTI disbursed for 2012, 2013 and 2014 MDAs did not exceed 33%[16] The government was paying 25 Francs to CDDs per person treated during the cost recovery era (households paid 100 Francs/person; later 10 Francs for people <15 years) as incentive. Since 2013 however, the government shifted the responsibility to the communities after owing CDDs for a number of years, and without thorough sensitization. Each community was asked to decide how to support their CDDs. The communities in turn pushed the responsibility to household members. The amount agreed to be paid to CDDs varied by community, it ranged from 0–500 Francs per household. Twenty (20) out of twenty-four (24) community heads reported that they have been informed to support their CDDs but 449 (47.5%) of 945 community members said they were not aware they had to give cash incentive to CDDs (Figs 5 and 6 respectively). Some health service personnel and CDDs did not trust that the communities can fully assume the responsibility. In their responses to how prepared their communities were to shoulder the responsibility, one of the community heads lamented that: *“… even when we had to support our CDDs to buy petrol into their motorbike to go and collect Ivermectin is difficult for us”*. Four (4) of the twenty-four (24) community heads reported they did not participate in the decision to support CDDs; they were informed through letters or their CDD. Though not many of the CDDs reported having received incentives (17 out of 40) most of them (34 out of 40) said they would like to continue as CDDs to serve their communities. Twenty-six (26) of them reported being involved in other health interventions like bed net distribution and immunization where they receive cash incentives.

#### Human resources

CDTI activities were steered at the regional level by the regional coordinator for NTDs with assistance from other staff members who functioned as supervisors during campaigns. At the health district level, activities were steered by the DMOs, CBHs and Focal Persons for NTDs and they were also assisted by other district health service personnel to train and supervise COCs during campaigns. COCs were in charge of CDTI at the health areas: they were responsible for approaching the communities, train and supervise CDDs and CSM facilitators, summarize census and treatment results and report on treatment. However, only some of the health centers had supporting staff in addition to COCs, usually a pharmacy and laboratory attendants. The work load was usually beyond what they can efficiently manage. Here is the view of one COCs: *“I think CDTI should be contracted out*, *the work is difficult and approaching the community is frustrating”*. Another COC lamented: *“Hummm*! *The work load is too high on me*, *I sometimes recruit some people from the community to assist me*. *Especially the people of the health committee to assist me in supervision and sometimes filling the registers*. *I cannot do it because I am working alone*. *[…]*. *Is too much*. *I am the only nurse in the health center”*. The COCs more often had to leave their stations, sometimes for days to attend meetings and workshops.

Annual distribution of Ivermectin begins with training of CDTI personnel. DMOs, CBHs and District Focal persons for NTDs are trained at the regional level. These officials then train COCs and other health area staff involved in CDTI in their respective health districts. After their training, COCs mobilize CDDs for training by sending letters to community heads. Training was done every year for CDTI staff. In 2014, 124 CDDs were trained for distribution in our 25 study communities with mean CDD population ratio = 1 CDD: 317 population (range: 194–464. The denominator further reduced when measured against number of operational CDDs). Number of CDDs trained together at health area levels ranged from 14 to 127 (mean ± SD = 51 ±32) with duration of training ranging from 4–7 hours (mean ± SD = 5.05 ± 1.09), as shown in Table 2. Most of the trainings were conducted at the health centers.

**Table 2:** Characteristics of CDD training for the past three distributions in our study area.

CDDs Training	Minimum	Mean ± SD	Maximum
No. times trained in past 3 yrs	3	3 ± 0.00	3
Number trained together	14	51 ± 32	127
Duration of training (hours)	4	5.05 ± 1.09	7
CDD-Population ratio	194	317 ± 88	464

Some CDDs were not permanently resident in the communities, others were either on their farms for most part of the day during farming seasons or they work outside the communities. Only 40 of 124 CDDs in our study communities were sampled after a second round visit. According to the South West Regional Delegation of Public Health (SWRDPH) database, the two projects in the region achieved 100% of targeted CDD recruitment each year for the three years. However, CDD attrition was a major challenge; it was 14% (2012), 11% (2013) and 12% (2014) of total CDDs trained in the region. The figures were higher in some health districts; 20% (2012), 13% (2013) and 20% (2014) for one particular Health District. CDD attrition was attributed to lack of incentive.

#### Distribution of Ivermectin

Distribution of Ivermectin was carried in June (2012); in October (2013) and in August (2014). Distribution follows training. After training of CDDs, they are given registers to conduct census of their communities, based on which supply of Ivermectin to each community is determined. According to the COCs, the programme experienced transport problems (usually during supervisions) every year for the three distributions (Mean ± SD = 1.58 ± 1.50). The COCs updated their target populations for the three distributions (Mean ± SD = 2.75 ± 0.89). The mean number of times supply of Ivermectin delayed was (Mean ± SD = 0.17 ± 0.58). Also, CDDs reported (Mean ± SD = 0.55 ± 0.93) as mean number of times they experienced shortage of drug during distribution and (Mean ± SD = 2.85 ± 0.58) as mean number of times they conducted census before distribution (Tables 3 and 4).

**Table 3:** The experiences of COCs with Ivermectin distribution over the past three years.

Variable	Minimum	Mean ± SD	Maximum
No. times experienced transport problem	0	1.58 ± 1.50	3
No. times supply delayed	0	0.17 ± 0.58	2
No. times target population updated	0	2.75 ± 0.89	3

**Table 4:** CDDs’ experience with Ivermectin distribution over the past three years.

Variable	Minimum	Mean ± SD	Maximum
No. times experienced shortage	0	0.55 ± 0.93	3
No. times conducted census	0	2.85 ± 0.58	3

#### Supervision of CDTI activities

Supervision was carried out on training at all levels and distribution in the communities. The mean number of times COCs were supervised was (Mean ± SD = 2.75 ± 1.06) and the mean number of times CDDs were supervised by a health workers during the three distributions was (Mean ± SD = 2.40 ± 1.01). The mean number of review meetings conducted on CDTI at the district level was (Mean ± SD = 2.00 ± 0.95). Table 5 gives details on supervision activities.

**Table 5:** Frequency of supervision and review meeting carried out over the past three years.

Variable	Minimum	Mean ± SD	Maximum
No. times COCs were supervised	1	2.75 ± 1.06	4
No. review meetings on CDTI at district level	1	2.00 ± 0.95	4
No. times CDDs were supervised by health worker.	0	2.40 ± 1.01	3

#### Monitoring CDTI activities

Community Self-monitoring (CSM) is the main mechanism by which CDTI activities at the community level should be monitored. The regional coordinator randomly selects 2–4 communities per health area for CSM. There was a budget for CSM from the external funding. The timing and allocation of funds for CSM was not reliable: CSM was not carried out in 2014 because the programme managers did not receive funds, one COC pointed out that monitors at community level ask for incentive. All twelve (12) COCs interviewed were very familiar with CSM; on the other hand, after thorough description of CSM to the twenty-four (24) community heads interviewed, only 2 admitted the process has ever been carried out in their communities (Fig 5). All twenty-four (24) community heads reported having not selected monitors. A CBH reported: *“I think CSM is not up to 50% good among the population due to monitors asking for incentive*”.

## Discussion

The goal of APOC was to establish community-led CDTI projects that are sustainable within twelve (12) years after start of the project in a particular jurisdiction [4]. This requires full participation of communities in the design of implementation strategies, sensitization of its members and mobilization of human and financial resources, CDTI works best by the principle of active social participation [1,9,10]. Exploring the operational challenges faced by CDTI projects in our study communities revealed that a top-down approach to planning of CDTI activities may have resulted in low community participation. As observed by Gyapong *et al*., active community participation fosters better adherence to treatment [17]. Active community participation could have guided the program to organize MDAs at periods when the community members are most available to optimally participate. Practices such as communities not selecting CDDs through general community elections has also been reported in other CDTI projects [9,14]. In another study, community-member participation in CDD selection was observed to be a positive predictor of adherence to treatment. [18].

Studies have demonstrated that when communities are informed and empowered, they are able to deliver health interventions that are more complex than Ivermectin MDA [19,20]. The inability of the health service to engage and thoroughly educate communities on their roles in the CDTI process therefore, constituted a huge implementation barrier. Hence, communities lacking knowledge of their roles regarding planning, resourcing and monitoring of the process, and perceiving CDTI as exclusive responsibility of the government after 16 years of MDA is blamable on lack of sensitization. Lack of sensitization may also have resulted in poor knowledge of the causes of onchocerciasis and the dynamics of treating using Ivermectin among community members and hence they may not have adhered optimally to treatment. It was important that the health service closely followed APOC’s guidelines for mobilizing communities for CDTI through series of meetings with the people in their respective communities instead of using letters [8]. In an APOC midterm evaluation conducted in Cameroon, implementers asserted that more health sensitization was needed to achieve the revised target of 80% therapeutic coverage; the report added that sensitization was more critical in the face of 25 severe adverse effects plus one death and 12 severe adverse effects plus two deaths in 2008 and 2009 respectively and also to remove strong suspicion among CDDs that health staffs were siphoning their cash incentives [21]. In the face of the current goal of eliminating onchocerciasis as a disease of public health concern by 2025, sensitization must also be strategized to actively identify systemic non adherers to be educated and properly treated since they may hold a reservoir of the infection in the population [22]. Again, sensitization through television and radio broadcastings, posters and fliers must be scaled up and augmented with home visits by CDDs to announce and educate household members on Ivermectin distribution.

Financial issues may have also affected the implementation of CDTI in the area. The government and other donor agencies must endeavor to meet their budgetary allocations to the programme to ensure smooth implementation. The subcontracting arrangements between funding partners possibly introduced complexities into funding procedures. The programme could have benefited from a more streamlined funding process that minimizes delays and ensures adequate funding for all aspects of implementation. The cost recovery approach adopted by Cameroon from the beginning of CDTI may have hampered the ability of health staff to persuade the communities to accept their ‘new responsibility’ of having to provide cash incentive to CDDs [22]. Clearly, inappropriate community support to CDTI derailed APOC’s objective of establishing a community-led distribution in our study area, possibly because the principles that foster community ownership, empowerment and sustainability were not applied [21,23–26]. It therefore would be helpful for the health services to engage the communities to explore all possible alternatives of providing incentives to implementers at the community level. Another issue that may have challenged CDTI activities here is inadequate health workers. Integration of NTD programmes has been generally reckoned as cost effective. However, this could mean increased work load for implementers at frontline health facility levels and unbearable costs to communities. Given the human resource limitations at the frontline health facilities in our study area, a health staff having to implement several health interventions including primary health care services, coupled with frequent travels for meetings and workshops posed serious challenges to the CDTI process [21]. Consequently, Inadequate health staff at the frontline health facility level negatively influenced the way communities were approached and sensitized, the quality of training given to CDDs and CSM facilitators, supervision and monitoring of distribution. Lack of close supervision of distribution by health staff was also attributed to human resource gap in another study carried out in some communities of the Mungo river drainage [27]. Inadequate health staff partly explain why instead of training CDDs and CSM facilitators in their own communities, they were trained together (both new and old) in large numbers at health centers. An interesting dimension to the human resource limitation was high CDD attrition. The midterm evaluation conducted in Cameroon before this study reported a better CDD: population ratio of 1:123 (1:86–1:300) [21], indicative that the situation may have gone worse. High CDD attrition and hence turnover necessitated annual selection and training of new CDDs for replacements. The high attrition may have been caused by CDDs not receiving cash incentive for CDTI work but receiving such on other interventions using the CDTI approach. Again, organizing MDAs during the peak of farming seasons may have been a key contributory factor to CDD attrition. This partly explains why most of the CDDs in our study communities were missed out during the data collection. The problem may have been further compounded by inability of health staff to clearly explain modes of CDD remuneration under the CDTI concept. However, some CDDs expressed willingness to continue their work in spite of lack of incentives. They expressed feeling important as they became involved in community-level decision making and are sometimes referred to as ‘doctors’ by community members. In the midterm evaluation report, health staff believed more training of implementers would be needed in order to achieve the revised coverage of 80% [21]. The inclusion of hypoendemic areas in treatment to meet current goal of WHO to eliminate ochocerciasis by 2025 implied training of more implementers to meet the increased workload.

Another possible setback faced by CDTI in these areas was the inability of the programme managers to carry out distribution in the dry months of the year. Initial meetings with communities, sensitization, training of CDDs and CSM facilitators, supervision and monitoring of distribution are activities that heavily depend upon efficient transportation. Most communities in these areas become hard to reach during the rainy season due to poor nature of roads. Fares usually double and health staff spend hours in transit or simply avoided these areas. Transportation challenges has been noted to have adversely affected CDTI operations in other jurisdictions [21,27]. These physical and health system effects created barriers to effective implementation of annual distributions and may have actually led to low therapeutic coverage of the population [28,29].

The key internal mechanism put in place to identify operational challenges with the CDTI is Community Self-Monitoring (CSM). However, CSM was not a common practice among the study communities. This was likely due to inability of the health service to mobilize well-motivated CSM facilitators and to develop strong partnership with the communities in this regard. Monitoring of CDTI activities is very key since programme failure would result in onchocerciasis recrudescence and hence rolling back of the socioeconomic and health gains made against the disease over the years [21,30]. Understanding the weaknesses of CDTI would be necessary in maintaining its relevance in the current NTD landscape.

The CDTI approach gave huge impetus to onchocerciasis control on the continent through increased geographic and therapeutic coverage, and hence the possibility of using it to deliver other health interventions has since been explored to some extent [2]. The approach thus looks promising to current NTD control efforts on the continent, more importantly, given the fact that most onchocerciasis endemic countries in Africa are also faced with serious health professional crisis. Especially, among the most affected, rural and poor communities. The use of CDDs and other community health workers therefore, would be crucial in bridging the health professional gaps of these countries. Again, the CDTI approach may become a key strategy in ESPEN’s integrated approach to controlling the five NTDs amenable to preventive chemotherapy (namely: onchocerciasis, lymphatic filariasis, schistosomiasis, soil transmitted helminthes and trachoma), given the fact that the approach proved efficient in delivering similar interventions, including distribution of Long-Lasting Insecticide-Treated Bed Nets, Vitamin A and home management of malaria [1,31]. Also, the CDTI approach holds the potential to become more relevant in delivering more other interventions in the near future. Following the discovery of thermostable dog rabies vaccine, studies are now ongoing to ascertain the prospects of a scaled-up community-led administration [32]. Further, the CDTI approach is in perfect harmony with international calls for inclusion of populations in the health decision making process. The objective to create a lean NTD control entity, as a result of which ESPEN was created, is most likely possible only at regional and country levels. ESPEN would need the contribution of the huge CDD work force within communities to be able to sustain the momentum and health gains achieved by APOC [31]. Finally, the approach would remain relevant into the foreseeable future because it is probably the most cost efficient. Prior to inception of CDTI, the World Bank, UNICEF, UNDP, WHO special programme for Research and Training in Tropical Diseases (TDR) and OCP jointly conducted a trial study in five countries across West, Central and Eastern Africa to compare the effectiveness of vertical campaigns to a community-directed approach. The study demonstrated that coverage, cost-effectiveness, community acceptance and empowerment were better for CDTI as compared to vertical campaigns [4]. Also, structural community participation prepares communities better for subsequence sustainability and continuation of intervention as opposed to vertical approaches.

### Limitations of this study

Since parasitological and entomological surveys were not done in this study, it is difficult to conclude that implementation challenges were responsible for the inability of CDTI to control onchocerciasis in the study area as expected. The recent parasitological and entomological studies conducted in our study area cited vector competence, favorable breeding conditions for the vector as possible factors responsible for the persisting transmission and high prevalence, though this paper did not compare coverage data from other parts of the country, its findings would likely be same for other endemic areas with similar transmission potentials and geographic characteristics. In such terrains, there would probably be a need for an additional intervention like vaccination when it becomes available, or moving from annual to biannual treatment. The current approach of Test and Treat being used in Cameroon may increase the health gains if it is followed comprehensively.

### Conclusion

Certain critical weaknesses existed in the implementation process of CDTI in our study area. This included weak community participation towards planning of CDTI activities, sensitization of community members, resource mobilization and monitoring of the process. This may have actually led to low adherence to Ivermectin treatment among community members.

Also, inadequate staff at the frontline health facilities, funding issues and transportation challenges derailed efforts of the health service towards implementing adequate training, supervision and monitoring of the process.

### Recommendations

Future studies should combine entomological, epidemiological, as well as data on the programme performance in order to identify and better explain the factors responsible for certain control outcomes. Also, it would be interesting to examine how closely figures for indictors such as therapeutic coverage, reported by CDTI projects reflect what is actually achieved in the populations.

The guidelines on engaging and mobilizing communities for CDTI activities must be comprehensively followed. This should reflect a change in perception of health staff of communities as mere beneficiaries of the process to an attitude that regards the importance of the roles of communities. The roles of communities should be clearly communicated to them, and they should be trained to totally assume those roles. Planning of CDTI activities must begin from community level, with increased community participation in the planning, supervision and monitoring of the process. Again, integration of CDTI with other primary health care deliveries must be quickly broadened to cover many interventions as possible. However, this must be done with caution in order not to dilute the CDTI concept.

## Supporting information

S1 STROBE Checklist Items included in this cross-sectional study. (DOC)

## Abbreviations

CDTI: Community-Directed Treatment with Ivermectin
NDGOs: Non-Governmental Developmental Organizations
APOC: African Programme for Onchocerciasis Control
CDDs: Community Drug Distributors
CSM: Community Self-Monitoring
ONCHOSIM: Onchocerciasis Transmission Elimination Simulation Model
MDA: Mass Drug Administration
TC: Treatment Coverage
NTDs: Neglected Tropical Diseases
CBH: Chiefs of Bureau Health
COC: Chiefs of Centers
SSI: Sightsavers International
HKI: Hellen Keller International
USAID: United States Agency for International Development
WHO: World Health Organization
DMO: District Medical Officer
SWRDPH: South West Regional Delegation of Public Health
ESPEN: Expanded Special Programme for Elimination of Neglected Tropical Diseases
UNICEF: United Nations International Children Emergency Fund
UNDP: United Nations Development Programme
TDR: WHO special programme for Research and Training in Tropical Diseases
OCP: Onchocerciasis Control Programme
